# 1D Lead Bromide Hybrids Directed by Complex Cations: Syntheses, Structures, Optical and Photocatalytic Properties

**DOI:** 10.3390/molecules29174217

**Published:** 2024-09-05

**Authors:** Ya-Qi Liu, Sen Huang, Ji-Dong Leng, Wei-Quan Lin

**Affiliations:** School of Chemistry and Chemical Engineering, Institute of Clean Energy and Materials, Guangzhou University, Guangzhou Higher Education Mega Center, No. 230 Wai Huan Xi Road, Guangzhou 510006, China; itsyaki@163.com (Y.-Q.L.); 15363886632@163.com (S.H.)

**Keywords:** organic–inorganic hybrid materials, transition metal complex cation, single-crystal structure, photocurrent response, photocatalytic activity, hydrogen evolution reaction

## Abstract

This study presents the synthesis, structural characterization, and evaluation of the photocatalytic performance of two novel one-dimensional (1D) lead(II) bromide hybrids, [Co(2,2′-bpy)_3_][Pb_2_Br_6_CH_3_OH] (**1**) and [Fe(2,2′-bpy)_3_][Pb_2_Br_6_] (**2**), synthesized via solvothermal reactions. These compounds incorporate transition metal complex cations as structural directors, contributing to the unique photophysical and photocatalytic properties of the resulting materials. Single-crystal X-ray diffraction analysis reveals that both compounds crystallize in monoclinic space groups with distinct 1D lead bromide chain configurations influenced by the nature of the complex cations. Optical property assessments show band gaps of 3.04 eV and 2.02 eV for compounds **1** and **2**, respectively, indicating their potential for visible light absorption. Photocurrent measurements indicate a significantly higher electron–hole separation efficiency in compound **2**, correlated with its narrower band gap. Additionally, photocatalytic evaluations demonstrate that while both compounds degrade organic dyes effectively, compound **2** also exhibits notable hydrogen evolution activity under visible light, a property not observed in **1**. These findings highlight the role of metal complex cations in tuning the electronic and structural properties of lead(II) bromide hybrids, enhancing their applicability in photocatalytic and optoelectronic devices.

## 1. Introduction

In the realm of materials science, the exploration and synthesis of organic–inorganic hybrid materials have captivated researchers because of their intriguing structural complexities and the unique properties that emerge from the interplay of their inorganic and organic components. Particularly, hybrid lead(II) halogenides have surfaced as a notable subclass, offering extensive functionalities that render them suitable for applications ranging from solar cells [1,2] to ferroelectric [3] and optical devices [4,5]. The synthesis of new multi-functional hybrid lead(II) halogenide materials has, therefore, become a focal point in the field, spurred by the drive to harness their potential in various technological domains [6,7].

Particularly, these materials have demonstrated noteworthy photocatalytic activities, encompassing both the degradation of organic pollutants [8,9] and photocatalytic hydrogen evolution reaction (HER) [10]. The unique electronic structure of these materials, tailored by their hybrid nature, allows for efficient light absorption and charge carrier management, enhancing their performance as photocatalysts. The photocatalytic capabilities of hybrid lead(II) halogenides in both pollutant degradation and hydrogen production underscore their potential for addressing environmental challenges and advancing renewable energy technologies [11].

The versatility of lead(II) ions in adopting diverse coordination geometries presents a rich tapestry of structural possibilities. From quadrangular pyramids to octahedra, the ability of lead(II) ions to form various condensed structures underpins the structural diversity and the multifaceted properties of these materials [12]. The use of organic cations as templating agents has traditionally guided the synthesis of numerous lead halogenide clusters and frameworks, contributing significantly to the understanding and expansion of this material class [13,14,15]. However, the introduction of transition metal (TM) complex cations as countercations represents a relatively unexplored avenue with the potential to unveil new hybrid materials characterized by novel structural motifs and enhanced functionalities [14,16,17,18].

One-dimensional (1D) hybrid lead(II) halogenides exhibit unique characteristics distinct from their three-dimensional (3D) counterparts, particularly in terms of stability, optics, and electrical properties [19,20,21,22]. The 1D structure, influenced by the linking mode of inorganic anions, exhibits pronounced local electronic states because of the strong quantum confinement effect, resulting in a wider band gap and greater exciton binding energy [3]. This quantum confinement enhances the material’s photophysical properties, leading to broadband emission with significant Stokes shift, attributed to self-trapped exciton states [23]. Additionally, 1D metal halogenides demonstrate improved moisture stability compared to their 3D analogs, which is crucial for applications in perovskite solar cells [24]. The unique assembly of metal halide octahedra in 1D structures, through corner-, edge-, or face-sharing, presents them as bulk assemblies of quantum wires at the molecular level, distinct from nanoscale morphological 1D materials.

This study introduces transition metal complex cations as a strategic approach to synthesizing new complex–inorganic hybrid lead(II) halogenide materials. By employing solvothermal reactions, we synthesize two unprecedented compounds, [Co(2,2′-bpy)_3_][Pb_2_Br_6_CH_3_OH] (**1**) and [Fe(2,2′-bpy)_3_][Pb_2_Br_6_] (**2**), showcasing 1D lead(II) bromide chains. The two compounds not only enrich the structural diversity of hybrid lead(II) halogenides but also offer a new perspective on their potential properties. The integration of transition metal complexes is posited to influence the electronic, optical, and magnetic characteristics of the resultant materials, potentially paving the way for advancements in their application in photocatalytic and optoelectronic devices.

In this paper, we report the solvothermal synthesis of the two new complex–inorganic hybrid compounds, accompanied by the investigations of their crystal structures, optical properties, photocurrent, and photocatalytic performance. Through this study, we aim to emphasize the significance of transition metal complex cations in the design and synthesis of hybrid lead(II) halogenides, highlighting their role in the evolution of this fascinating material class.

## 2. Results and Discussion

### 2.1. Structural Analysis

Single-crystal X-ray diffraction measurement revealed that compound **1** crystallizes in the monoclinic space group *P*2_1_/*n* (No. 14). Each asymmetric unit contains a [Co(2,2′-bpy)_3_]^2+^ complex cation and a repeating unit [Pb_2_Br_6_CH_3_OH]^2−^ of the lead bromide anion. The Co-N bond lengths in the complex range from 2.114(4) to 2.149(5) Å (Table S1, Figure S1a, Supplementary Materials), comparable to many previously reported Co^II^ complexes [8,25,26]. Compound **2** crystallizes in the monoclinic space group *P*2_1_/*c* (No. 14). In each asymmetric unit, a [Fe(2,2′-bpy)_3_]^2+^ complex cation and a repeating unit [Pb_2_Br_6_]^2−^ of the 1D lead bromide chain are present. The Fe-N bond lengths of 1.943(4) to 1.977(5) Å also fall within the same range as reported low-spin Fe^II^ structures (Table S4, Figure S1b) [27,28,29,30].

**Figure 1:**
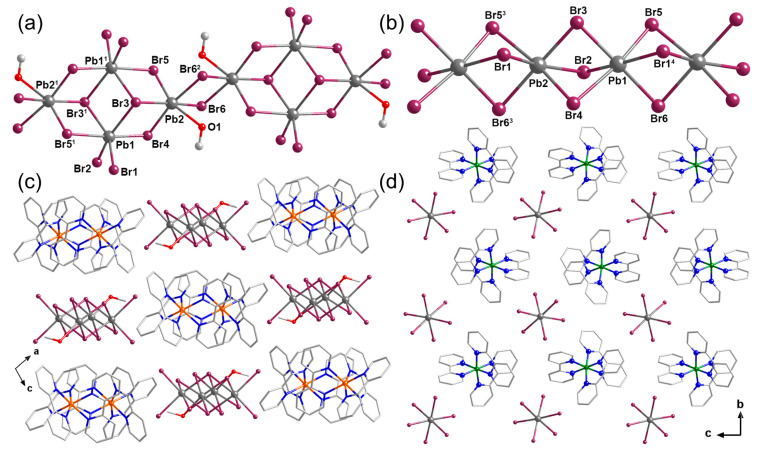
The crystal structures of the compounds **1** and **2**. (**a**) The 1D [Pb_2_Br_6_MeOH]^2−^ chain of **1**. (**b**) The 1D [Pb_2_Br_6_]^2−^ chain of **2**. (**c**) The packing diagram of compound **1** along the *b* axis. (**d**) The packing diagram of compound **2** along the *a* axis. Hydrogen atoms were omitted for clarity. Color code: Pb, gray; Br, plum; Co, orange; Fe, olive; N; blue; O, red; C, light gray. Symmetry code: ^1^ 2 − *x*, 2 − *y*, 1 − *z*; ^2^ 2 − *x*, 1 − *y*, 1 − *z*, ^3^ −1 + *x*, *y*, *z*; ^4^ 1 + *x*, *y*, *z*.

The structures of the lead bromide anions are both 1D chains for compounds **1** and **2** while possessing obviously different structural characteristics. For **1**, the 1D lead bromide chain is constructed through edge-sharing [PbX_6_] octahedrons (Figure 1a). The Pb–Br bond distances of 2.7638(6)–3.4043(6) Å (Table S1) and neighboring Br–Pb–Br(or O) angles of 69.49(11)–103.589(19) Å (Table S2) indicate the distorted nature of the octahedrons. Interestingly, in many reported hybrid lead(II) halogenides, such an edge-sharing structural characteristic often leads to double or triple chains [31]. In our case, the terminal coordinated methanol molecule led to the formation of a 1D chain composed of the rhombic structural unit [Pb_4_Br_12_MeOH_2_]^4-^ (Figure S2).

For compound **2**, the 1D lead bromide chain is a normal face-sharing single chain constructed through the repeating unit [Pb_2_Br_6_]^2−^ [24,31]. The 1D chains are then separated by the complex cations, forming the assembled structure (Figure 1c for **1** and Figure 1d for **2**). For both compounds, no obvious π–π stacking and hydrogen bonding interactions can be observed in the structure. Therefore, weak supramolecular interactions, such as C-H···π and anion–π, can play an important role in the formation of the stacked structure [13]. The shortest Pb···Pb distance of adjacent Pb-Br chains is 9.79 Å for **1** and 10.51 Å for **2**. The powder X-ray diffraction patterns of the two compounds are identical to the simulated result from the single-crystal structure (Figure S3).

The displacement between adjacent Pb centers represents lattice distortion, impacting the broadband/narrowband emission of self-trapped excitons found in materials with soft crystal lattices and electron–phonon coupling. Therefore, a set of distortion descriptors is useful for correlating the structures of organic–inorganic hybrid metal halide compounds with their emission properties [5]. Distortion of the octahedral PbBr_6_^4−^ building blocks in the inorganic lattice is assessed using bond length distortion (*Δ*_oct_), octahedral angle variance (*σ*_oct_), and the closest intra-chain Pb-Pb distance between adjacent Pb-Br octahedra. The results indicate that the distortion of the PbBr_6_^4-^ octahedral of **1** is larger than that of **2**.

Comparison with two reported compounds [31] containing 1D [PbBr_3_]^−^ single chains (Table 1) reveals that the bond length distortion and octahedral angle variance of compounds **1** and **2** are similar to the reported ones. However, the closest intra-chain Pb-Pb distance in compound **1** is longer, while that in compound **2** is shorter. The shorter intra-chain Pb-Pb distance in compound **2** suggests compression in the vertical direction of the chain, which can lead to stronger electron–phonon coupling.

Notably, X.-W. Lei et al. reported a series of lead bromide hybrids based on the Co-bpy complex, which were also obtained by solvothermal reactions but under different temperatures [8]. In addition, their structures were quite different from those of compound **1** in that there are no solvent molecules coordinating with the lead bromide anions. The compound [Co(2,2′-bpy)_3_]_2_{[Co(2,2′-bpy)]_3_Pb_7_Br_24_} features a 0D isolated {[Co(2,2′-bpy)]_3_Pb_7_Br_24_}^4−^ cluster. The compounds [Co(2,2′-bpy)_2_Br][PbBr_3_] and [Co(2,2′-bpy)_3_][Pb_3_Br_9_] consist of a 1D [PbBr_3_]^−^ single chain and [Pb_3_Br_9_]^3−^ double chain, respectively. While [Co(2,2′-bpy)_3_][Pb_5_Br_13_]·CH_3_CN features a 2D [Pb_5_Br_13_]^3−^ layer. The Lei group also reported two isomorphic silver lead halide hybrids [Co(2,2′-bpy)_3_]AgPb_2_I_7_ and [Fe(2,2′-bpy)_3_]AgPb_2_Br_7_, which feature 1D metal-halide chains as well [29].

On the other hand, the combination of [Co/Fe(2,2′-bpy)_3_]^2+^ complexes with silver halobismuthates [25], iodoargentates [32,33,34,35], cuprous bromides [36], and iodoplumbates [37] have also been reported. These results demonstrate the versatile template functions of the complexes in the synthesis of hybrid metal halogenides.

### 2.2. Syntheses

The solvothermal synthesis method is a versatile and powerful technique in materials science, renowned for its ability to yield a diverse array of materials with unique structures and properties. One of its principal advantages lies in the precise control it offers over reaction conditions, such as temperature, pressure, and solvent environment, which are pivotal in directing the crystal growth and phase purity of the resultant compounds [38].

In the context of our research, the distinction in reaction temperatures between the synthesis of compound **1** (160 degrees Celsius) and the series of lead bromide hybrids reported by X.-W. Lei et al. [8] (140 degrees Celsius) underscores the sensitivity of solvothermal synthesis to temperature variations. Particularly for compound **2**, despite numerous reports on [Fe(2,2′-bpy)_3_]-based lead(II) halide hybrids, the combination of this simple complex cation with the common lead bromide single chain in a compound remains unprecedented. The increase in temperature in our synthesis likely influenced several factors critical to the material’s formation, including reaction kinetics, solvent coordination, and the thermodynamic stability of the resulting compound. We hypothesize that the elevated temperature promoted enhanced coordination between methanol solvent molecules and lead ions, contributing to the thermodynamic stabilization of compound **1**. The ability to control such parameters is invaluable in materials chemistry, where the precise manipulation of synthesis conditions can lead to the discovery of materials with tailored functionalities.

Based on the simple solution experiments, the solubilities of the powder samples of both compounds in water are less than 0.05 mg mL^−1^. This low solubility can be attributed to the enhanced moisture stability of 1D metal halogenides [24] and the thermodynamic stability of the compounds.

### 2.3. Optical Properties

**Figure 2:**
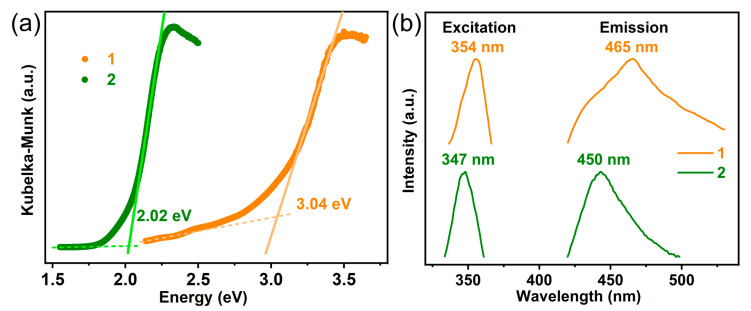
(**a**) UV-vis absorption spectra of **1** (orange) and **2** (olive). (**b**) Excitation (short wavelength region) and emission (long wavelength region) spectra at room temperature for **1** (orange) and **2** (olive).

The optical absorption band gaps (*E*_g_) of the two compounds were determined by UV-vis reflectance spectroscopy on polycrystalline samples, and raw data were converted using the Kubelka–Munk function (Figure 2a) [39]. The optical band gaps estimated by extrapolating the edge of the absorption band to the linear part for **1** and **2** are 3.04 and 2.02 eV, respectively. The results indicate their possible semiconductor nature and the existence of direct transitions. The band gaps of the two compounds, especially the 2.02 eV of **2**, are significantly narrower than many 1D hybrid lead bromides directed by organic cations, such as the single 1D chain (DBU)PbBr_3_ (*E*_g_ = 3.48 eV) [40], as well as the double chain (2,6-lutidine)PbBr_3_ (*E*_g_ = 3.61 eV) and (2-phenylpyridine)PbBr_3_ (*E*_g_ = 3.44 eV) [31,41,42,43].

The incorporation of transition metal complexes [Co(2,2′-bpy)_3_]^2+^ and [Fe(2,2′-bpy)_3_]^2+^ likely plays a crucial role in modifying the electronic structure. The diverse d–d transitions of these photosensitive TM complex cations contribute to the lower conduction band levels, resulting in a red shift of the absorption edge [29]. Such a band gap aligns with the requirements for materials used in light-harvesting applications, suggesting that the two compounds can be promising candidates for the development of new optoelectronic devices, where precise control over electronic properties is paramount for enhancing performance.

To further characterize the optical properties of the compounds, steady-state photoluminescence (PL) measurements were performed on their polycrystalline samples. Compounds **1** and **2** exhibited emissions maxima at ca. 465 and 450 nm, respectively (Figure 2b, *λ*_ex_ = 354 and 347 nm, respectively). Such PL emissions are similar to those of many previously reported compounds based on [TM(2,2′-bpy)_3_]^2+^ complexes and can be assigned to the emission of ligand-to-metal charge transfer [28]. At room temperature, the free 2,2′-bpy ligand exhibited weak emission at 530 nm in its solid state. The observed blue shifts and enhanced luminescence relative to free 2,2′-bpy were likely due to ligand coordination with metal ions. This coordination increased ligand rigidity and minimized energy loss through radiationless decay from the excited state of the intra-ligand emission [44]. Notably, the emission of compound **1** was significantly broader than that of compound **2**. This broadening could be attributed to two possible reasons. Firstly, the paramagnetism and strong spin-orbit coupling of Co²⁺ ions in compound **1** could cause the mixing and splitting of electronic state energy levels, resulting in multiple closely spaced energy levels. Transitions between these levels lead to a broadened emission spectrum [28]. Secondly, the greater distortion of the PbBr₆^4^⁻ octahedra in compound **1** might lead to stronger self-trapped excitons, thereby contributing to broadband emissions [45].

The PL emission of the anions is not observable in the spectrum. This can be attributed to the relatively low photoluminescence quantum yield (PLQY) of the 1D lead bromide chains (normally lower than 3%), which, in turn, is due to the nonradiative decay resulting from their low rigidity [31,45]. For comparison, the PLQYs of compounds **1** and **2** are 11.2% and 14.5%, respectively. Because of the similar PL behaviors of the two compounds, we speculate that their different band gaps originate from the different structures of their lead bromide components.

### 2.4. Photocurrent Measurements

**Figure 3:**
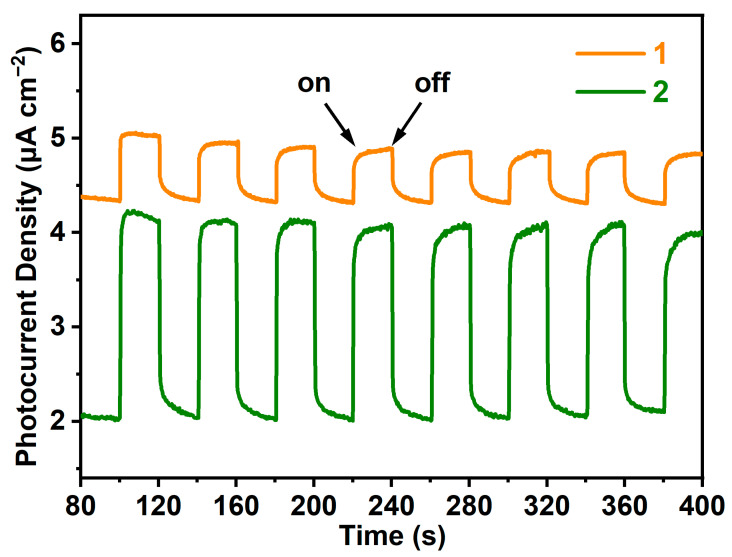
The photocurrent density of **1** (orange) and **2** (olive) under a xenon lamp mimicking sunlight. The curve of **1** is translated along the vertical axis to avoid overlapping.

The photocurrent response properties of compounds **1** and **2** were tested in a KCl solution using a classic three-electrode configuration and exhibited photocurrent densities of ca. 0.66 and 2.18 μA cm^−2^ (Figure 3), respectively. This finding is pivotal, as the photocurrent response is a critical indicator of the photogenerated electron and hole separation efficiency, which, in turn, reflects the photovoltaic and photocatalytic activity of the material [46]. Similar photocurrent response have also been studied in some high-performance metal halides, such as [Ag_2_I_2_(phen)] [47], {[Nd_2_(dpdo)(DMF)_14_](Ag_12_I_18_)}, {[La(dpdo)(DMF)_6_](Bi_2_I_9_)_2_} [48], [Ni(phen)_3_]Pb_2_I_6_·CH_3_CN [49], [Co(bipy)_3_]_2_Pb_8_I_21_ [37], [Ni(bipy)_3_]AgBiI_6_ [30], and [Zn(bipy)_3_]_2_Ag_2_BiI_6_(I)_1.355_(I_3_)_1.645_ [17]. Unfortunately, the photocurrent response property of the closely related compound [Co(2,2′-bipy)_3_]Pb_3_Br_9_ and [Co(2,2′-bipy)_3_]Pb_5_Br_13_·CH_3_CN [8] have not been reported.

Under identical experimental conditions, compound **2** exhibits significantly higher photocurrent density compared to compound **1**, corresponding to the previously mentioned narrower band gap of **2**. This is because materials with narrower band gaps, which allow for efficient absorption of visible light, can possess enhanced light-harvesting capabilities [24]. In this study, the narrower band gap of **2** enables more efficient absorption of visible light, leading to a higher generation of charge carriers. Additionally, the observed stronger current density under visible light in compound **2** can also suggest a more efficient separation and transport of charge carriers (electrons and holes). 

On the other hand, the robust photocurrent response and stability of the two compounds, even after multiple on/off cycles, highlight the excellent charge carrier transfer capacity and durability of these materials. Furthermore, the ability to maintain photocurrent response without significant decay aligns with observations in other high-performance metal halides, demonstrating the relevance and promise of our synthesized compound in the field of photoelectric materials [1].

### 2.5. Photodegradation Reactions of RhB

In the context of escalating concerns about water pollution due to the release of non-degradable organic compounds, photocatalysis emerges as a potent, environmentally benign solution. Employing solar light to drive the degradation of these pollutants, photocatalysis utilizes photogenerated electrons and holes to break down harmful organics into harmless molecules [18,50]. For instance, compounds with narrow band gaps have shown enhanced photocatalytic activities under visible light, effectively degrading various organic pollutants like rhodamine B (RhB), methyl orange (MO), and crystal violet (CV). Photocatalytic reactions primarily depend on the ability of the catalyst to generate and separate charge carriers effectively, a process enhanced in compounds with favorable electronic structures [9]. Moreover, similar studies have highlighted the influence of factors such as the structural characteristics, band gaps of the photocatalysts, and the intensity and duration of light exposure on the photocatalytic outcomes. 

**Figure 4:**
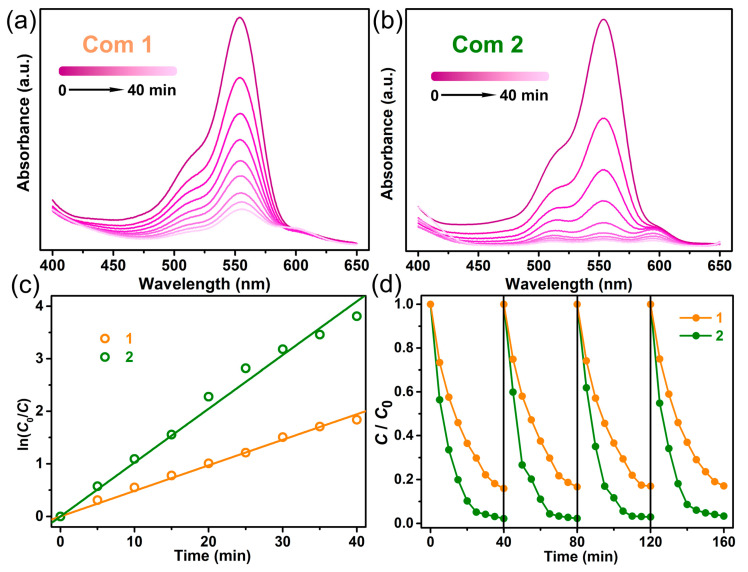
Time-dependent absorption spectra (**a**,**b**), the linear relationship of ln(*C*_0_/*C*) with reaction time (**c**), and the recyclability (**d**) of the photodegradation reactions of RhB solutions over compounds **1** and **2**. The solid lines in subfigure (**c**) are the fitting results using the equation ln(*C*_0_/*C*) = *kt*.

**Figure 5:**
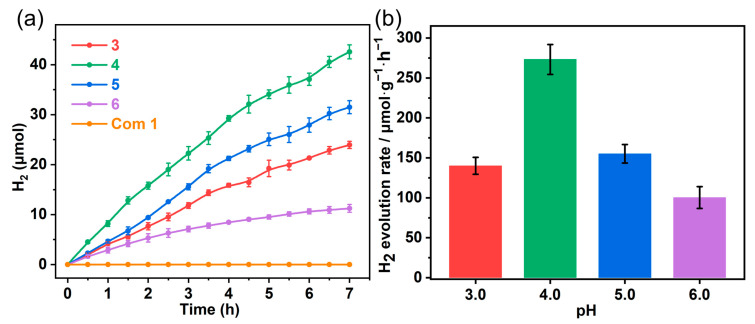
(**a**) Plots of photocatalytic hydrogen production versus time for compounds **1** (orange) and **2** in ascorbic acid solutions with different pH values. (**b**) Initial rates (μmol g^−1^ h^−1^) of photocatalytic hydrogen evolution for **2** at different pH values.

Encouraged by the photocurrent response property of the title compounds, their photodegradation efficacies were investigated using RhB as a model pollutant in an aqueous solution at room temperature. The concentration changes of RhB were monitored by checking the maximum absorption wavelength changes at 554 nm in UV-vis absorption spectra (Figure 4a,b). The evolution of *C*/*C*_0_ versus irradiation time is shown in Figure 5d, where *C*_0_ and *C* are the initial and instantaneous organic dye concentrations of organic dyes, respectively. Remarkably, both compounds exhibited high degradation efficiency, achieving a reduction in RhB of 84.1% for compound **1** and 97.7% for compound **2** within 40 min. Again, compound **2** displays higher degradation efficiency because of its narrower band gap. Given the first-order kinetic reaction of photodegradation, we determined the rate constants using the equation ln(*C*_0_/*C*) = *kt*, where *k* represents the rate constant (Figure 4c). The calculated *k* values are 0.049 min^−1^ for **1** and 0.10 min^−1^ for **2**, all significantly higher than that for PbBr_2_ (0.002 min^−1^). Such photocatalytic activities are comparable to other recent findings in the field of complex cation-templated halometallate hybrids [8,17,25,26,30,35,51]. 

To check the photochemical stability of the compounds, we further tested the stability and reusability by collecting and reusing the same photocatalyst for four cycles (Figure 4d). After four cycles, the final photocatalytic ratios over the initial ratios were maintained at 98.6% for **1** and 98.8% for **2**. The XRD patterns at the end of the repeated experiments are almost identical to those of the as-prepared samples (Figure S3). The results indicate not only the robustness of the compounds but also their suitability for repeated use without significant degradation in performance. This contrasts with other materials where structural degradation or a decrease in activity may occur after several cycles. For instance, similar studies have indicated that while compounds exhibit initial high activity, their structural stability under photocatalytic conditions can vary, affecting long-term performance [10].

### 2.6. Photocatalytic Hydrogen Evolution

TM complexes are also known for their redox-active properties and can be effective in photocatalytic applications. Combining the appropriate band gaps of the two compounds, they could possess photocatalytic HER activities [52]. The two compounds were then evaluated under visible light irradiation (*λ* > 420 nm) at 15 °C with ascorbic acid (1 M) as a sacrificial electron donor but without any co-catalyst and photosensitizer. Under the same experimental conditions, compound **2** was able to exhibit considerable photocatalytic HER activity, while compound **1** did not (Figure 5a). To investigate the optimal conditions for H_2_ production, we optimized the pH values of the aqueous solutions. As shown in Figure 5a, after 7 h of photocatalysis, the amounts of the produced H_2_ are 23.9, 42.6, 31.5, and 11.2 μmol for pH values of 3.0, 4.0, 5.0, and 6.0, respectively. The initial HER rate of 273 ± 18 μmol·g^−1^·h^−1^ at pH = 4.0 is higher than those at other pH values. The result suggests that the photocatalytic HER activity of compound **2** at a pH of 4.0 is the highest. This is because the pH value not only manifests the proton concentration of the medium but also affects the electron-donating ability of ascorbic acid [53] as well as the charge carrier dynamics of the photocatalyst [54]. 

The completely different photocatalytic HER activities of the two compounds can be attributed to the following three reasons. Firstly, with a band gap of 3.04 eV (equal to ca. 408 nm), compound **1** primarily absorbs UV light, which constitutes a smaller fraction of the solar spectrum, while the narrower band gap of **2** (2.02 eV, 614 nm) allows for efficient absorption of visible light and results in a higher generation of charge carriers necessary for photocatalytic reactions, thereby enhancing the photocatalytic activity. Secondly, the stronger current density observed in compound **2** under visible light indicates a more efficient separation and transport of charge carriers (electrons and holes). Efficient charge carrier dynamics reduce recombination losses and increase the availability of electrons for HER. Thirdly, as the transition metal centers and the structures of the 1D anionic chains of the two compounds are different, their different band edge positions can also influence the HER activities. The conduction band minimum being more negative relative to the hydrogen evolution potential accelerates the effective reduction of protons to hydrogen [55]. 

To further characterize the stability of these compounds, we conducted field-emission scanning electron microscopy (SEM) analyses for the fine-ground powder samples and the samples after the HER experiments (Figure S5 for **1** and Figure S6 for **2**). According to SEM images, the dimensions of the samples were on a micrometer scale. For both compounds, the SEM images revealed that there were no significant changes in the microstructural features, such as particle size and surface texture. This indicates that the two compounds exhibited excellent structural stability during the HER process. Moreover, the XRD patterns of the compounds after the HER experiments were also collected (Figure S3). The results further verified the stability of these compounds, where the obtained patterns were almost identical to those of the as-prepared samples.

## 3. Materials and Methods

### 3.1. General Remarks

All reagents and solvents for synthesis were commercially available and used as received without further purification. The water used in this study was purified via the Milli-Q technique (18.2 MΩ). All air-sensitive experiments were carried out with standard Schlenk techniques under a dry argon atmosphere. pH values of all solutions were tested using a Mettler Toledo pH meter (S210). The C, N, and H microanalyses were performed using an Elementar Vario EL Cube elemental analyzer (Langenselbold, Germany). The powder XRD patterns were determined with a Bruker D8 X-ray diffractometer (Billerica, MA, USA, CuK*α*) at room temperature. Simulated powder patterns were calculated by Mercury software (2023.3.0) using the crystallographic information file (CIF) from the single-crystal X-ray experiment. The microstructure and morphology of the compounds were obtained by field emission scanning electron microscopy (SEM, TESCAN MIRA LMS, Brno, Czech Republic) and transmission electron microscopy (TEM, JEOL JEM-2100F, Tokyo, Japan).

### 3.2. Syntheses

**Synthesis of [Co(2,2′-bpy)_3_][Pb_2_Br_6_CH_3_OH] (1)**

CoCl_2_·6H_2_O (23.8 mg, 0.1 mmol), 2,2-bpy (46.9 mg, 0.3 mmol), PbBr_2_ (110.1 mg, 0.3 mmol), and NaBr (30.9 mg, 0.3 mmol) in 10 mL methanol were sealed in a 25 mL Teflon-lined, stainless-steel vessel, heated at 160 °C for 72 h, and then cooled slowly to room temperature at 5 °C/hour. Light-yellow, block-shaped crystals were obtained (yield ca. 65% based on Co). Elemental analyses yielded the following (calc: found): C_31_H_28_Br_6_CoN_6_OPb_2_: C 25.62: 25.97, N 5.78:5.60, H 1.94: 1.97.

**Synthesis of [Fe(2,2′-bpy)_3_][Pb_2_Br_6_] (2)**

FeSO_4_ (15.2 mg, 0.1 mmol), 2,2-bpy (46.9 mg, 0.3 mmol), PbBr_2_ (110.1 mg, 0.3 mmol) and NaBr (30.9 mg, 0.3 mmol) in 10 mL methanol were sealed in a 25 mL Teflon-lined, stainless-steel vessel, heated at 160 °C for 72 h, and then cooled slowly to room temperature at 5 °C/hour. Red plate-shaped crystals were obtained (yield ca. 55% based on Fe). Elemental analyses yielded the following (calc: found): C_30_H_24_Br_6_FeN_6_Pb_2_: C 25.41: 25.64, N 5.93: 6.20, H 1.71: 1.83.

### 3.3. X-ray Structure Determination

Suitable crystals were selected and mounted on a suitable support on an Rigaku XtaLAB Synergy R, DW system (Tokyo, Japan), HyPix diffractometer. The crystals were kept at a steady *T* = 149.99(10) K during data collection. The structures were solved with the ShelXT [56] structure solution program using the Intrinsic Phasing solution method and using Olex2 (1.5) [57] as the graphical interface. The models were refined with version 2017/1 of ShelXL 2017/1 using least-squares minimization [56]. 

X-ray crystallographic files for the structures were deposited in the Cambridge Crystallographic Data Centre (CCDC) with no. 2340431 (**1**) and 2351252 (**2**). The data can be obtained free of charge via www.ccdc.cam.ac.uk/conts/retrieving.html (or from the Cambridge Crystallographic Data Centre, 12 Union Road, Cambridge CB21EZ, UK; fax (+44)1223-336-033 or e-mail deposit@ccdc.cam.ac.uk).

### 3.4. Optical Properties

Optical diffuse-reflectance spectra of powder samples were obtained at room temperature with a Shimadzu UV-3150 spectrometer (Kyoto, Japan) using BaSO_4_ as the reference of 100% reflectance. Absorption (*α*/*S*) data were calculated from the reflectance using the Kubelka–Munk function *α*/*S* = (1 − *R*)^2^/2*R*, where R is the reflectance and α and S are the absorption and scattering coefficients, respectively. Steady-state emission and excitation spectra were measured on an Edinburgh FLS-980 Fluorescence spectrometer (Livingston, UK) equipped with a visible photomultiplier tube (PMT) and a NIR PMT upon excitation with a continuous Xenon lamp. All the excitation and emission spectra were corrected for the instrumental functions.

### 3.5. Photocurrent Measurements

Time-dependent photocurrent measurements for the compounds under investigation were conducted using a Chenhua CHI760E electrochemical workstation (Shanghai, China). During these measurements, an Ag/AgCl electrode served as the reference, a Pt wire was used as the auxiliary electrode, and the device in question acted as the working electrode. The working electrode was prepared using a typical drop-coating method. Initially, 5 mg of microcrystalline powder was dispersed in a mixture of ethanol and Nafion solution (ratio 475:25), followed by sonication. This mixture was then gradually applied to a pre-treated ITO glass surface and left to dry at ambient temperature. Illumination during the experiments was provided by a 300 W xenon lamp mimicking sunlight, while a 0.1 M solution of KCl in water was the chosen electrolyte for the process (a schematic diagram of the device is shown in Figure S4).

### 3.6. Photodegradation Properties

The photocatalytic activities of the as-prepared compounds were assessed under visible light irradiation using RhB as a model organic dye. A quantity of 30 mg of compound **1** powder was dispersed in 50 mL of a 1 × 10^−5^ mol L^−1^ RhB aqueous solution. This suspension was stirred continuously in the dark for 10 min to ensure the attainment of adsorption/desorption equilibrium between the photocatalyst and the dye. The suspension was irradiated using a 300 W xenon (Xe) lamp equipped with a cut-off filter to eliminate ultraviolet and infrared wavelengths, thereby permitting only visible light (400 nm < λ < 780 nm) to pass through. The lamp was positioned to ensure uniform light distribution across the reaction vessel. During the irradiation process, 4 mL samples were periodically withdrawn from the reaction cell at predetermined time intervals. Each sample was immediately centrifuged to separate the photocatalyst particles from the dye solution. The concentration of RhB in each supernatant sample was determined by measuring the maximum absorption at 554 nm using a Shimadzu UV-2550 UV/vis spectrophotometer (Kyoto, Japan). The degradation ratio of RhB was calculated by comparing the intensity of the absorption band before and after exposure to visible light irradiation.

### 3.7. Photocatalytic Hydrogen Evolution

In a typical experiment, 30 mg of the fine-ground sample of the compound was added into 30 mL 1 M ascorbic acid solution and sonicated for 20 min. And, the pH was adjusted to a certain value by the addition of saturated sodium hydroxide [52]; after that, the suspension was transferred to a 270 mL photocatalytic glass reactor and bubbled with argon gas with stirring for 30 min. Then, the 270 mL photocatalytic glass reactor was transferred to the top-irradiation reactor and irradiated under a 300 W xenon lamp (Perfect Light PLS-SXE 300, Farmers Branch, TX, USA) with a 420 nm cut-off filter. The reaction temperature was maintained at 15 °C through the cooling water system. The amount of H_2_ evolution was determined via gas chromatograph (GC-2018, SHIMADZU Analytical Instrument, Kyoto, Japan) with a 5 Å molecular sieves column. The initial rate of photocatalytic H_2_ evolution was calculated based on the early one-hour hydrogen evolution reaction.

## 4. Conclusions

This study successfully demonstrated the synthesis and characterization of two novel one-dimensional lead(II) bromide hybrids, compounds **1** and **2**, utilizing transition metal complex cations as structural directors. Compound **1**, although limited in its ability to participate in photocatalytic hydrogen evolution, showed commendable performance in the degradation of organic pollutants, highlighting its potential in environmental remediation. Compound **2**, in contrast, not only excelled in pollutant degradation but also demonstrated hydrogen evolution activity under visible light, attributed to its narrower band gap and effective charge carrier dynamics. These activities underline the critical role of structural and electronic customization in enhancing the photocatalytic efficiency of hybrid materials.

In conclusion, the integration of transition metal complexes into the structure of lead(II) halogenides offers a promising route to tailor material properties for specific functional applications, enhancing both the versatility and efficacy of these hybrid materials. Future studies will focus on expanding their utility to other forms of renewable energy production and environmental decontamination.

## Figures and Tables

**Table 1:** Distortion descriptors of **1**, **2,** and two reported compounds consisting of 1D [PbBr_3_]^−^ single chain.

Compound	*Δ*_oct_	*σ*_oct_	Intra-Chain Pb-Pb Distance (Å)
Pb1 (**1**)	5.95 × 10^−3^	9.85	4.47
Pb2 (**1**)	1.17 × 10^−3^	10.05	
Pb1 (**2**)	2.13 × 10^−3^	8.29	3.89
Pb2 (**2**)	1.71 × 10^−3^	8.29	
(2,4-LD)PbBr_3_	3.28 × 10^−3^	9.36	3.95
(2-MP)PbBr_3_	3.35 × 10^−2^	10.46	4.01

## Data Availability

The original contributions presented in the study are included in the article and supplementary materials, further inquiries can be directed to the corresponding authors.

## Supplementary Materials

The following supporting information can be downloaded at https://www.mdpi.com/article/10.3390/molecules29174217/s1, Table S1: Crystal data and structure refinement for **1** and **2**. Table S2: Selected Bond Lengths (Å) for **1**. Table S3: Selected Bond Angles (°) for **1**. Table S4: Selected Bond Lengths (Å) for **2**. Table S5: Selected Bond Angles (°) for **2**. Figure S1: The crystal structure of the complex cation [Co(2,2-bpy)_3_]^2+^ (for compound **1**, a) and [Fe(2,2-bpy)_3_]^2+^ (for compound **2**, b). Displacement ellipsoids set at the 50% probability level and hydrogen atoms omitted for clarity. Color code: Co, orange; Fe, olive; N; blue; C, light gray. Figure S2: The structure of the 1D [Pb_2_Br_6_MeOH]^2−^ chain showing the structural unit [Pb_4_Br_12_MeOH_2_]^4−^, which is highlighted by the blue-gray diamonds; Figure S3: Simulated (black) and experimental powder X-ray diffraction patterns of the as-prepared sample (blue) and the sample after photocatalysis (red) for compound **1** (a) and **2** (b). Figure S4: Schematic diagram of the device for the measurement of the photocurrent. Figure S5: SEM images of compound **1** before (a and b) and after (c and d) the HER experiments. Figure S6: SEM images of compound **2** before (a and b) and after (c and d) the HER experiments. Figure S7. TEM image of compound **2** after the HER experiments.
