# Supplementary material for: 1D Lead Bromide Hybrids Directed by Complex Cations: Syntheses, Structures, Optical and Photocatalytic Properties
Source: Molecules. 2024 Sep 5;29(17):4217. doi: 10.3390/molecules29174217 (PMC11397344; doi:10.3390/molecules29174217)
Supplement: Supplementary file 1 [file molecules-29-04217-s001.zip › molecules-3025957-supplementary.pdf]

Supplementary Material

1D Lead bromide hybrids directed by complex cations: syntheses, structures, optical and photocatalytic properties

Ya-Qi Liu, Sen Huang, Ji-Dong Leng*, Wei-Quan Lin*

School of Chemistry and Chemical Engineering/Institute of Clean Energy and Materials, Guangzhou University No. 230 Wai Huan Xi Road, Guangzhou Higher Education Mega Center, Guangzhou 510006 (P. R. China)

* Corresponding author.

E-mail addresses: jidong.leng@gzhu.edu.cn (J.-D. Leng) ; linwquan@gzhu.edu.cn (W.-Q. L.)

Table S1. Crystal data and structure refinement for **1** and **2**.

**Compound**	**1**	**2**
Formula	$C_{31}H_{27}Br_6CoN_6OPb_2$	$C_{30}H_{24}Br_6FeN_6Pb_2$
$D_{calc.}$/ g cm^{-3}	2.527	2.548
μ/mm^{-1}	15.542	15.987
Formula Weight	1452.35	1418.24
Colour	clear light yellow	red
Shape	block	plate
Size/mm^3	0.06×0.05×0.05	0.20×0.10×0.03
T/K	149.99(10)	149.99(10)
Crystal System	monoclinic	monoclinic
Space Group	$P2_1/n$	$P2_1/c$
a/Å	12.7464(4)	7.8422(2)
b/Å	12.5514(3)	20.9211(5)
c/Å	24.3218(7)	22.6076(7)
α/°	90	90
β/°	101.161(3)	94.612(3)
γ/°	90	90
V/Å^3	3817.54(19)	3697.16(17)
Z	4	4
Z'	1	1
Wavelength/Å	0.71073	0.71073
Radiation type	Mo K_α	Mo K_α
Θ_{min}/°	2.299	2.606
Θ_{max}/°	27.000	25.998
Measured Refl.	36426	29624
Independent Refl.	8288	7274
Reflections with I > 2(I)	7120	5709
R_{int}	0.0467	0.0949
Parameters	425	406
Restraints	0	18
Largest Peak	2.381	4.794
Deepest Hole	-2.197	-3.664
GooF	1.069	1.071
wR_2 (all data)	0.0945	0.1893
wR_2	0.0913	0.1784
R_1 (all data)	0.0414	0.0829
R_1	0.0340	0.0651

Table S2. Selected Bond Lengths (Å) for **1**.

Atom	Atom	Length/Å
Pb1	Br1	2.8890(6)
Pb1	Br2	2.7638(6)
Pb1	Br3[1]	3.4043(6)
Pb1	Br3	3.1218(6)
Pb1	Br4	2.9941(6)
Pb1	Br5[1]	3.3833(7)
Pb2	Br3[1]	3.0911(6)
Pb2	Br4	3.0024(6)
Pb2	Br5	2.8018(7)
Pb2	Br6	2.9964(6)
Pb2	Br6[2]	3.1000(6)
Pb2	O1	2.920(5)
Co1	N1	2.149(5)
Co1	N2	2.128(5)
Co1	N3	2.124(5)
Co1	N4	2.135(5)
Co1	N5	2.114(4)
Co1	N6	2.131(5)

[1]2-x,2-y,1-z; [2]2-x,1-y,1-z

Table S3. Selected Bond Angles (°) for **1**.

Atom	Atom	Atom	Angle/°
Br1	Pb1	Br3	152.964(17)
Br1	Pb1	Br3[1]	98.375(17)
Br1	Pb1	Br4	103.589(19)
Br1	Pb1	Br5[1]	78.050(16)
Br2	Pb1	Br1	90.447(19)
Br2	Pb1	Br3	90.926(19)
Br2	Pb1	Br3[1]	161.171(18)
Br2	Pb1	Br4	84.702(19)
Br2	Pb1	Br5[1]	101.051(18)
Br3	Pb1	Br3[1]	88.703(15)
Br3	Pb1	Br5[1]	75.189(16)
Br4	Pb1	Br3	103.424(19)
Br4	Pb1	Br3[1]	77.088(16)
Br4	Pb1	Br5[1]	174.054(17)
Br5[1]	Pb1	Br3[1]	97.049(15)
Br3[1]	Pb2	Br6[2]	103.369(16)
Br4	Pb2	Br3[1]	82.039(16)
Br4	Pb2	Br6[2]	169.50(2)
Br5	Pb2	Br3[1]	84.659(19)
Br5	Pb2	Br4	98.28(2)
Br5	Pb2	Br6	91.40(2)
Br5	Pb2	Br6[2]	91.239(19)
Br5	Pb2	O1	159.69(11)
Br6	Pb2	Br3[1]	170.238(17)
Br6	Pb2	Br4	89.720(17)
Br6	Pb2	Br6[2]	85.607(17)
O1	Pb2	Br3[1]	105.48(11)
O1	Pb2	Br4	100.51(11)
O1	Pb2	Br6[2]	69.49(11)
O1	Pb2	Br6	81.09(11)
Pb1	Br3	Pb1[1]	91.297(15)
Pb2[1]	Br3	Pb1[1]	94.736(15)
Pb2[1]	Br3	Pb1	99.911(17)
Pb1	Br4	Pb2	105.827(19)
Pb2	Br5	Pb1[1]	100.10(2)
Pb2	Br6	Pb2[2]	94.394(17)
N2	Co1	N1	77.27(18)
N2	Co1	N4	92.13(18)
N2	Co1	N6	93.25(18)
N3	Co1	N1	90.87(18)
N3	Co1	N2	164.68(17)
N3	Co1	N4	77.99(18)

**Atom**	**Atom**	**Atom**	**Angle/°**
N3	Co1	N6	99.38(17)
N4	Co1	N1	89.85(18)
N5	Co1	N1	97.75(18)
N5	Co1	N2	95.75(18)
N5	Co1	N3	95.44(18)
N5	Co1	N4	170.07(18)
N5	Co1	N6	77.45(18)
N6	Co1	N1	169.01(18)
N6	Co1	N4	96.12(17)

[1]2-x,2-y,1-z; [2]2-x,1-y,1-z

Table S4: Selected Bond Lengths (Å) for **2**.

Atom	Atom	Length/Å
Pb1	Br1[1]	3.2527(15)
Pb1	Br2	2.8709(16)
Pb1	Br3	3.0038(14)
Pb1	Br4	3.2009(13)
Pb1	Br5	2.9080(13)
Pb1	Br6	3.0297(14)
Pb2	Br1	2.9007(14)
Pb2	Br2	3.1882(16)
Pb2	Br3	2.9897(14)
Pb2	Br4	2.8959(12)
Pb2	Br5[2]	3.2117(13)
Pb2	Br6[2]	3.0661(14)
Fe1	N1	1.943(10)
Fe1	N2	1.948(10)
Fe1	N3	1.961(10)
Fe1	N4	1.977(11)
Fe1	N5	1.965(10)
Fe1	N6	1.968(10)

[1]1+x,+y,+z; [2]-1+x,+y,+z

Table S5: Selected Bond Angles (°) for **2**.

Atom	Atom	Atom	Angle/°
Br2	Pb1	Br1[1]	172.00(4)
Br2	Pb1	Br3	85.88(4)
Br2	Pb1	Br4	80.32(4)
Br2	Pb1	Br5	92.60(4)
Br2	Pb1	Br6	91.30(4)
Br3	Pb1	Br1[1]	97.10(4)
Br3	Pb1	Br4	80.31(4)
Br3	Pb1	Br6	177.09(4)
Br4	Pb1	Br1[1]	107.47(4)
Br5	Pb1	Br1[1]	79.81(4)
Br5	Pb1	Br3	93.92(4)
Br5	Pb1	Br4	171.12(4)
Br5	Pb1	Br6	85.50(4)
Br6	Pb1	Br1[1]	85.61(4)
Br6	Pb1	Br4	99.93(4)
Br1	Pb2	Br2	170.02(4)
Br1	Pb2	Br3	90.05(4)
Br1	Pb2	Br5[2]	80.61(4)
Br1	Pb2	Br6[2]	91.40(4)
Br2	Pb2	Br5[2]	104.64(4)
Br3	Pb2	Br2	80.71(4)
Br3	Pb2	Br5[2]	100.47(4)
Br3	Pb2	Br6[2]	178.55(4)
Br4	Pb2	Br1	95.50(4)
Br4	Pb2	Br2	80.17(4)
Br4	Pb2	Br3	85.72(4)
Br4	Pb2	Br5[2]	172.64(4)
Br4	Pb2	Br6[2]	94.05(4)
Br6[2]	Pb2	Br2	97.84(4)
Br6[2]	Pb2	Br5[2]	79.87(3)
Pb2	Br1	Pb1[2]	78.40(3)
Pb1	Br2	Pb2	81.04(4)
Pb2	Br3	Pb1	82.30(4)
Pb2	Br4	Pb1	80.45(3)
Pb1	Br5	Pb2[1]	78.97(3)
Pb1	Br6	Pb2[1]	79.52(3)
N1	Fe1	N2	82.3(4)
N1	Fe1	N3	90.0(4)
N1	Fe1	N4	98.6(4)
N1	Fe1	N5	176.1(4)
N1	Fe1	N6	95.3(4)
N2	Fe1	N3	96.9(4)
N2	Fe1	N4	178.0(4)
N2	Fe1	N5	95.1(4)
N2	Fe1	N6	88.1(4)
N3	Fe1	N4	81.3(4)
N3	Fe1	N5	93.2(4)
N3	Fe1	N6	173.1(4)
N5	Fe1	N4	84.1(4)
N5	Fe1	N6	81.7(4)
N6	Fe1	N4	93.6(4)

[1]1+x,+y,+z; [2]-1+x,+y,+z

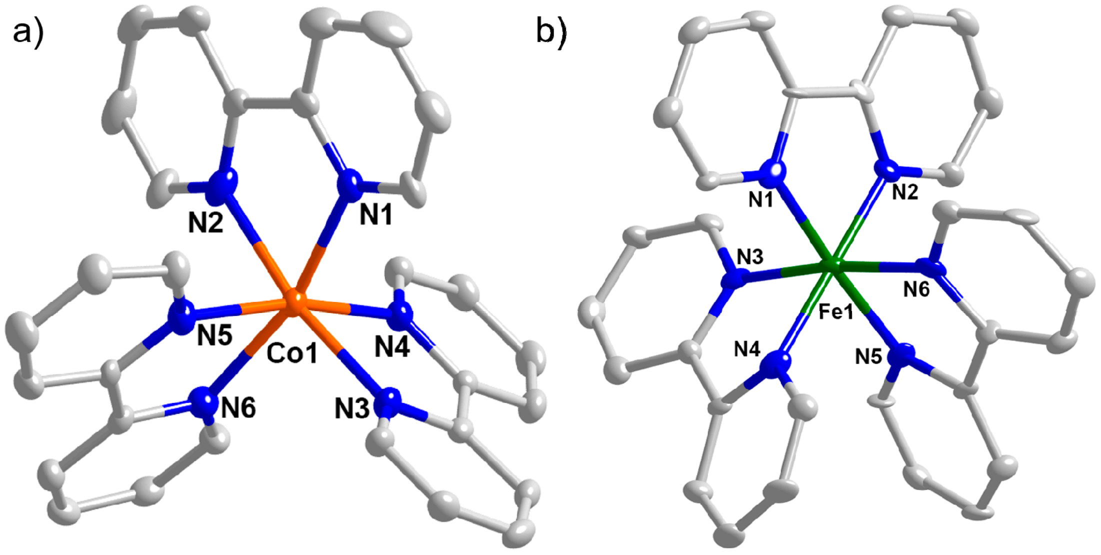

Figure S1. The crystal structure of the complex cation $[Co(2,2\text{-bpy})_3]^{2+}$ (for compound **1**, a) and $[Fe(2,2\text{-bpy})_3]^{2+}$ (for compound **2**, b). Displacement ellipsoids set at the 50% probability level and hydrogen atoms omitted for clarity. Color code: Co, orange; Fe, olive; N; blue; C, light gray.

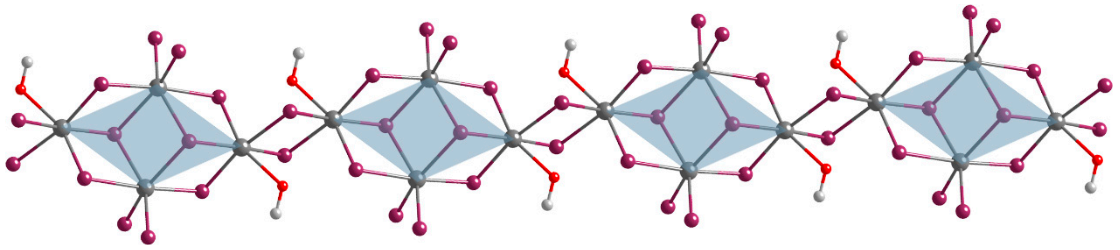

Figure S2. The structure of the 1D $[Pb_2Br_6MeOH]^{2-}$ chain showing the structural unit $[Pb_4Br_{12}MeOH_2]^{4-}$, which is highlighted by the blue gray diamonds.

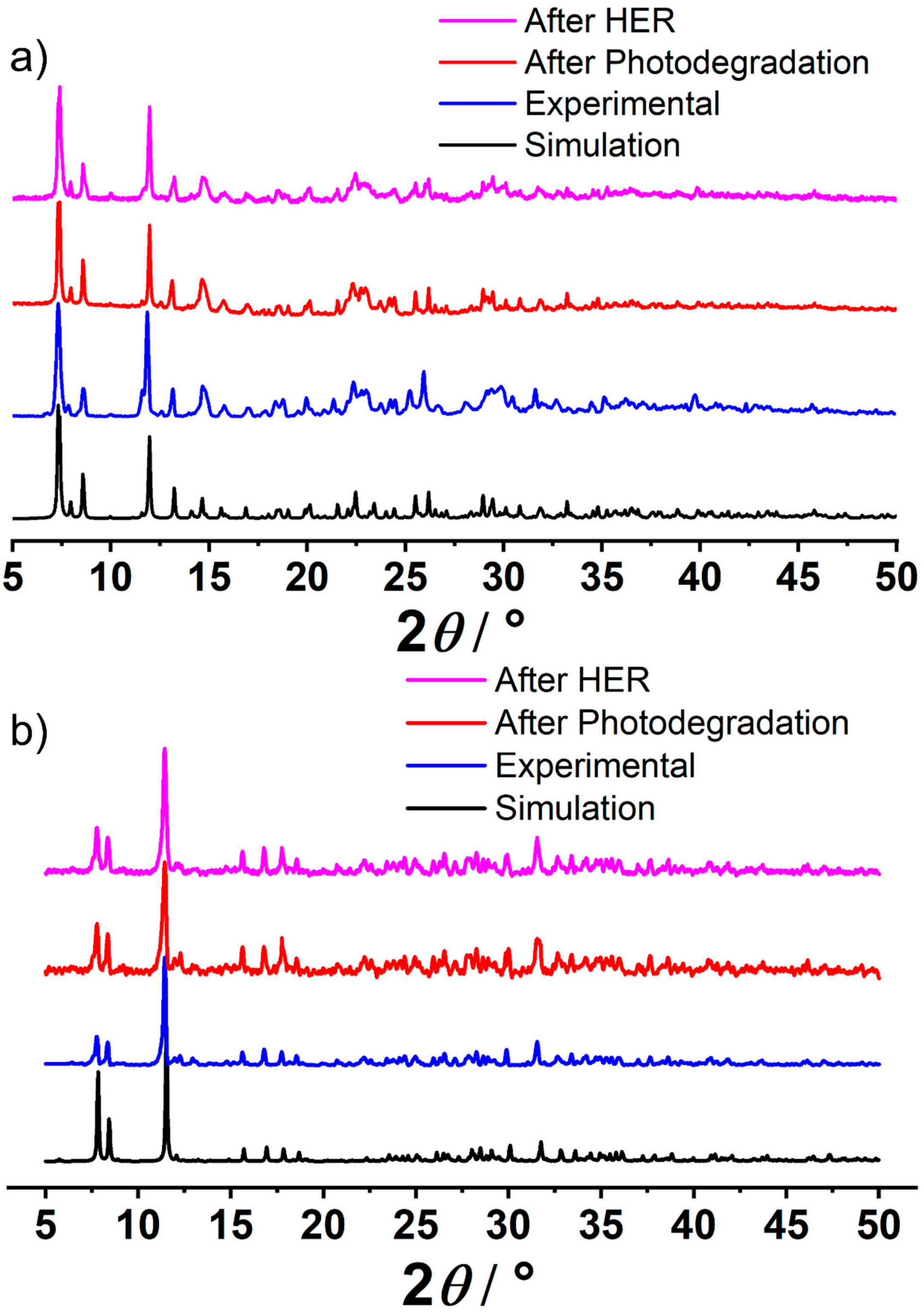

Figure S3. Powder X-ray diffraction patterns for compound **1** (a) and compound **2** (b): simulated patterns: black; as-prepared sample: blue; Sample after photodegradation: red; sample after HER: magenta.

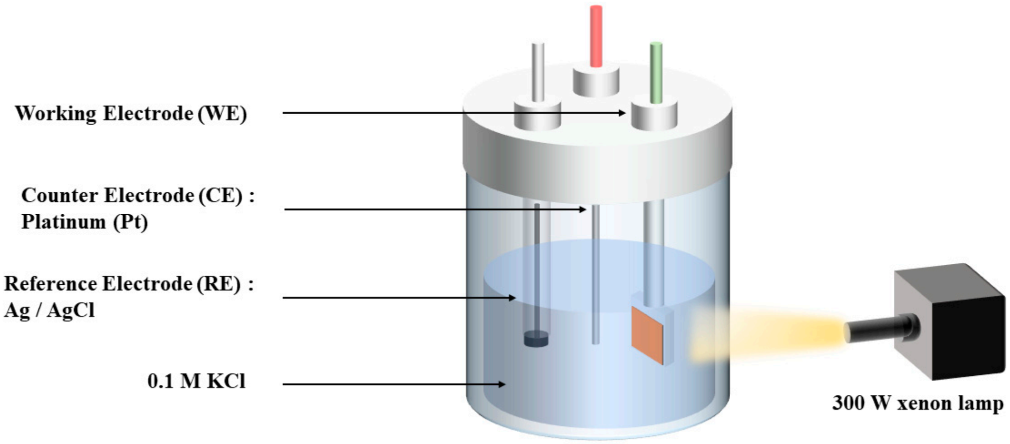

Figure S4. Schematic diagram of the device for the measurement of the photocurrent.

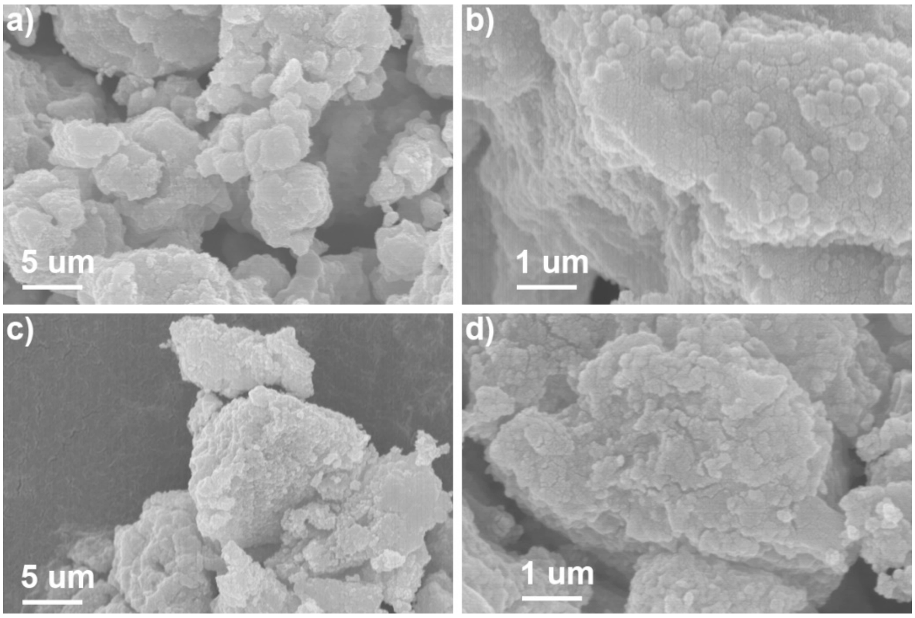

Figure S5. SEM images of compound **1** before (a and b) and after (c and d) the HER experiments.

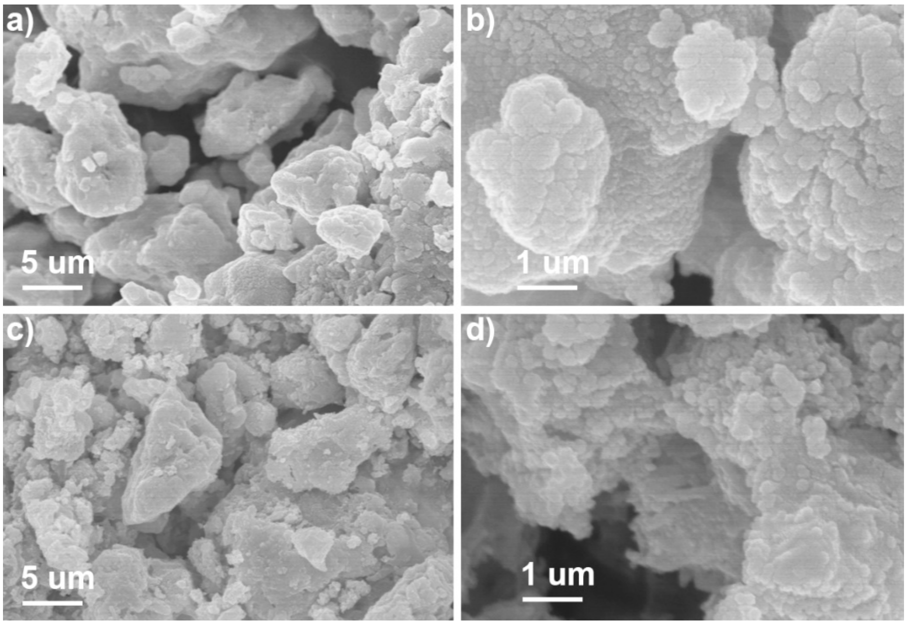

Figure S6. SEM images of compound **2** before (a and b) and after (c and d) the HER experiments.

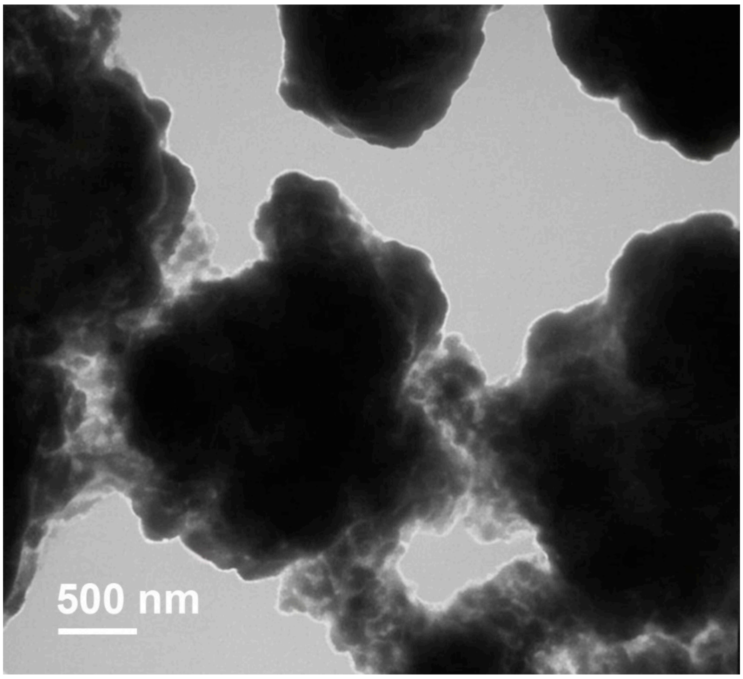

Figure S7. TEM image of compound **2** after the HER experiments. However, since the sample sizes are much larger than the nanoscale, the electron beam could not penetrate them.
